# Inodorous Iodoform

**Published:** 1883-11

**Authors:** 


### Inodorous Iodoform.

Tbe peculiar odor of iodoform is found to be well masked by the addition of attar of rose, one minim to the drachm, or of essence of rose geranium, three to four minims to the drachm. The clinic room gets to smell like a florist's shop.—*Polyclinic.*

